# Clinical Trial of Allogeneic Mesenchymal Stem Cell Therapy for Chronic Active Antibody-Mediated Rejection in Kidney Transplant Recipients Unresponsive to Rituximab and Intravenous Immunoglobulin

**DOI:** 10.1155/2021/6672644

**Published:** 2021-02-10

**Authors:** Tae Hyun Ban, Sua Lee, Hyung Duk Kim, Eun Jeong Ko, Bo-Mi Kim, Kyoung-Woon Kim, Byung Ha Chung, Chul Woo Yang

**Affiliations:** ^1^Division of Nephrology, Department of Internal Medicine, Eunpyeong St. Mary's Hospital, College of Medicine, The Catholic University of Korea, Seoul 03312, Republic of Korea; ^2^Division of Nephrology, Department of Internal Medicine, Seoul National University Hospital, Seoul 03080, Republic of Korea; ^3^Division of Nephrology, Department of Internal Medicine, Seoul St. Mary's Hospital, College of Medicine, The Catholic University of Korea, Seoul 06591, Republic of Korea; ^4^Transplant Research Center, Department of Internal Medicine, Seoul St. Mary's Hospital, College of Medicine, The Catholic University of Korea, Seoul 06591, Republic of Korea; ^5^Convergent Research Consortium for Immunologic Disease, Seoul St. Mary's Hospital, The College of Medicine, The Catholic University of Korea, Seoul 06591, Republic of Korea; ^6^Research & Development Center, OncoInsight, Seoul 06373, Republic of Korea

## Abstract

Clinical trials of biologic agents for chronic active antibody-mediated rejection (CAMR) in kidney transplant recipients (KTRs) have been disappointing. We performed a clinical trial of mesenchymal stem cell (MSC) treatment in KTRs with CAMR unresponsive to rituximab and intravenous immunoglobulin. This study was a phase 1 clinical trial to confirm patient safety. Two patients with CAMR unresponsive to rituximab and intravenous immunoglobulin were included. Each patient received allogeneic MSCs for 4 cycles (1 × 10^6^ cells/kg every other week) via the peripheral vein in the distal arm. We observed adverse events and renal function for 6 months after the final MSC infusion and analyzed changes in immunomodulatory parameters in the peripheral blood between the start of treatment and 3 months after the final MSC infusion. There were no serious adverse events during the study period. Renal function was stable during MSC treatment but gradually decreased between the final MSC infusion and the study endpoint (patient 1: creatinine levels ranged from 3.01 mg/dL to 7.81 mg/dL, patient 2: 2.87 mg/dL to 3.91 mg/dL). In peripheral blood sample analysis between the start of treatment and 3 months after the final MSC infusion, there were similar trends for immunomodulatory markers. Our study showed that there were no serious adverse events for six months after allogeneic MSC treatment in KTRs with CAMR refractory to rituximab and intravenous immunoglobulin, but further studies need to define the efficacy of MSC treatment in CAMR.

## 1. Introduction

Chronic active antibody-mediated rejection (CAMR) in kidney transplant recipients (KTRs) is a major cause of late kidney allograft loss. CAMR-related allograft failure recently occurred in nearly half of KTRs [1]. Therefore, therapeutic strategies, such as rituximab and bortezomib administration, have been used for years to overcome CAMR [2]. Unfortunately, studies including randomized controlled trials have revealed disappointing results [3, 4]. However, many clinicians have identified mesenchymal stem cells (MSCs) as a novel therapy. In previous studies, MSC treatment was shown to be effective in various kidney diseases [5]. These effects may originate from the potential of MSCs to differentiate into diverse cell types, including osteoblasts, chondrocytes, adipocytes, endothelial cells, and other organ cells. Although MSC therapy is expected to be a novel promising treatment for CAMR in kidney transplantation (KT), the therapeutic mechanism of MSCs is not fully understood. In emerging evidence, the core functions of MSCs as a therapy for many diseases may be regeneration and immunomodulation [6–9]. With regards to the therapeutic mechanisms of MSCs, their effects on KTRs are expected to produce favorable outcomes, such as minimization or withdrawal of immunosuppressive agents, decreased infectious complications, and reduced incidence of rejection.

As expected, the application of MSCs in KT is mainly conducted as an alternative to induction agent therapy and minimization of maintenance immunosuppressants [6, 7]. In a pilot study on KT, KTRs with MSC infusion had greater renal function than those without infusion during the five- to seven-year follow-up period [10, 11]. Thereafter, the addition of MSCs to conventional maintenance immunosuppressive agents suggests the possibility of reducing acute rejection after KT [12]. The largest clinical trial to date involved 105 KTRs [13]. The study reported faster organ regeneration, a lower rate of cellular rejection, and a decreased risk of opportunistic infection in MSC-treated patients. In regard to acute rejection, infusion of 2 MSC doses improved rejection as determined by follow-up allograft biopsy [14]. Finally, a study of a rat model reported the possibility of a therapeutic effect of MSCs on chronic allograft nephropathy [15]. On the basis of this rationale, we planned a clinical trial to confirm the safety of MSCs in KTRs with CAMR. In addition, based on previous studies [16, 17], we evaluated changes in T cells to determine the effects of MSCs.

## 2. Materials and Methods

### 2.1. Patient Enrollment and Study Protocol

This study was a phase 1, single-center, open-label pilot study to confirm safety in patients receiving MSC treatment. The inclusion criteria of the study were patients between 20 and 65 years of age who had CAMR confirmed by allograft biopsy within 6 months before MSC infusion and were unresponsive to the first-line treatment in our center. The first-line treatment for CAMR in our center was combined therapy with rituximab and intravenous immunoglobulin [18, 19]. In addition, steroid pulse therapy was done with intravenous methylprednisolone 500 mg per day for the first two days, followed by oral prednisolone 30 mg per day. After the first-line treatment, allograft function was assessed with serum creatinine levels and estimated glomerular filtration rate (eGFR) at monthly basis, and unresponsiveness was defined as a patient who failed to show improvement in renal function until 2 months after first-line treatment. Exclusion criteria included patients with hepatitis B, hepatitis C or HIV, a history of cardiovascular disease within 6 months, a New York Heart Association (NYHA) class 3 or 4 condition, a past history of malignancy, pregnancy (if female), and multiorgan transplantation. Finally, two KTRs who were diagnosed with CAMR by allograft biopsy were enrolled in this study. The therapeutic protocol of this study was approved by Seoul St. Mary's Hospital (KC18CESI0009) and the Korean Food and Drug Administration (KFDA) (KFDA 31810). The enrolled patients agreed to the study protocol and signed an informed consent form. This study was conducted in accordance with the Declaration of Helsinki.

**Figure 1:**
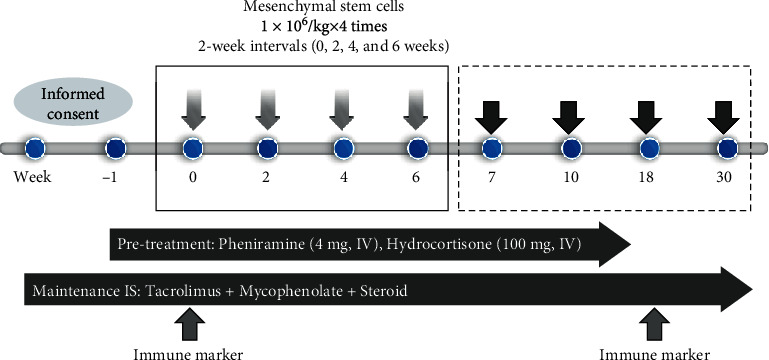
Clinical protocol for mesenchymal stem cell treatment of kidney transplant recipients with chronic active antibody-mediated rejection.

The therapeutic protocol of this study is illustrated in Figure 1. Each patient received allogeneic MSCs by intravenous injection via the peripheral vein in the distal arm. The MSC treatment protocol consisted of 4 cycles of 1 × 10^6^ cells/kg administered every other week. After the final cycle of MSC treatment, the patients were examined at 1 week, 1 month, 3 months, and six months. During the entire period of the study, maintenance immunosuppressive therapy was prescribed at the same dosage used during the pretreatment period as a mandatory recommendation of the KFDA. It consisted of tacrolimus (target trough blood levels of 3-8 ng/mL), mycophenolate, and a low-dose corticosteroid.

The patients were educated to report any discomfort symptoms and signs and then monitored for adverse events for 6 months after the final MSC infusion. We evaluated vital signs, any symptoms and signs, hematological and urinary parameters, tacrolimus trough levels, cytomegalovirus and BK virus PCR test results, patient survival, infectious complications, and other adverse events at every patient visit. Adverse events were assessed according to Common Terminology Criteria for Adverse Events version 5.0. We also analyzed peripheral blood samples from each patient to confirm changes in parameters of T cell-related immunomodulation between the start of MSC treatment and 3 months after the final MSC infusion. Parameters associated with B cells were excluded owing to the initiation of this study within 3 months after rituximab-based treatment as the first-line treatment for CAMR in our center.

### 2.2. Mesenchymal Stem Cells Derived from Human Bone Marrow

Human bone marrow-derived MSCs (Catholic MASTER Cells) were obtained from the Catholic Institute of Cell Therapy (CIC, Seoul, Korea). The Catholic MASTER Cells were allogeneic MSCs certified by the KFDA. The cells were obtained by human bone marrow aspiration from the iliac crest of healthy donors between the ages of 20 and 55 years. The process was approved by the Institutional Review Board of Seoul St. Mary's Hospital (approval numbers KIRB-00344-009 and KIRB-00362-006). Bone marrow aspirated from the donor was collected and sent to the CIC under good manufacturing practice (GMP) conditions. The CIC was responsible for the isolation, expansion, and quality control of allogeneic MSCs. Detailed information on the Catholic MASTER Cells was reported in a previous study [20]. Briefly, under conditions of bacterial sterility, mycoplasma sterility, and a low endotoxin level (<3 EU/mL) in the GMP-compliant facility, cells were expanded and tested for multilineage differentiation and cell-surface antigens.

### 2.3. Isolation and Flow Cytometric Analysis of Peripheral Blood Mononuclear Cells

Peripheral blood was collected from each patient at the start of the study and 3 months after the final infusion of allogeneic MSCs. Peripheral blood mononuclear cells (PBMCs) were prepared from heparinized blood by Ficoll ± Hypaque (GE Healthcare, PA) density-gradient centrifugation. Cell culture was performed as described previously [21]. In brief, a cell suspension was adjusted to a concentration of 10^6^ cells/mL in RPMI 1640 medium supplemented with 10% fetal calf serum, 100 U/mL penicillin, 100 mg/mL streptomycin, and 2 mM L-glutamine. The cell suspension (1 ml) was dispensed in 24-well multiwell plates (Nunc, Roskilde, Denmark).

### 2.4. Flow Cytometry

For the samples used for *in vitro* experiments, flow cytometric analysis was performed after collection of PBMCs. In the *in vitro* study, cells were stained with monoclonal antibodies: anti-CD4-PE/Cy7 (RPA-T4, IgG1, BioLegend, San Diego, CA), anti-CD8-APC (SK1, IgG1, *κ*, BD Biosciences, San Diego, CA), anti-CD161-APC (HP-EG10, IgG1, eBioscience, San Diego, CA), anti-CD45RA-FITC (HI100, IgG2b, *κ*, BD Biosciences), anti-CD28-PE (CD28.2, IgG1, *κ*, eBioscience), anti-CD57-FITC (TB01, IgM, eBioscience), anti-CD127-FITC (A7R34, IgG2a, *κ*, eBioscience), and anti-CD25-APC (M-A251, IgG1, *κ*, BD Biosciences). Staining for chemokine receptors was performed using the following mouse monoclonal antibodies (mAbs; all produced by BD Biosciences): anti-CCR4-PE (1G1, IgG1), anti-CCR6-APC (11A9, IgG1), and anti-CCR7 (3D12, IgG2a, *κ*).

For cytokine detection at the single-cell level, PBMCs were stimulated with 50 ng/mL phorbol myristate acetate (PMA) and 1 *μ*g/mL ionomycin in the presence of GolgiStop (BD Biosciences) for 4 hours. For intracellular staining, the cells were washed, fixed, permeabilized, and stained with the following monoclonal antibodies: anti-interleukin- (IL-) 17-PE (eBio64CAP17, IgG1, *κ*, eBioscience), anti-interferon (IFN)-*γ*-FITC (4S. B3, IgG1, *κ*, eBioscience), anti-IL-4-APC (11B11, IgG1, *κ*, eBioscience), and anti-Foxp3-PE (PCH101, IgG2a, *κ*, eBioscience). Isotype controls were used to monitor for nonspecific binding. Cells were measured using a FACSCalibur flow cytometer and FlowJo software.

### 2.5. Statistical Analysis

Statistical analyses were performed using SPSS 24.0 software. Changes in continuous variables according to MSC treatment were compared using the Wilcoxon signed-rank test. A *p* value < 0.05 was considered significant.

## 3. Results

### 3.1. Clinical Study

**Figure 2:**
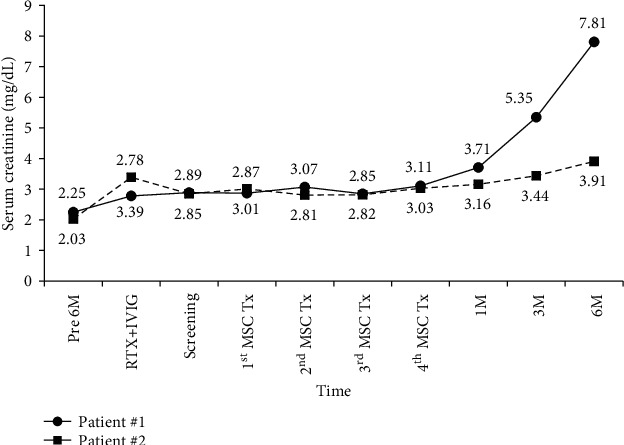
Renal function over time from 6 months before MSC treatment to 6 months after the final infusion. M: months; RTX: rituximab; IVIG: intravenous immunoglobulin; MSCs: mesenchymal stem cells; Tx: treatment.

The KT profile and posttransplant history of each patient are summarized in Table 1. CAMR in the patients was diagnosed with allograft biopsy owing to increased serum creatinine levels at 6 months in patient #1 and at 2 months in patient #2 before MSC treatment. Ahead of MSC infusion, the patients received first-line therapy for CAMR. However, the renal function of the patients did not improve (Figure 2). Accordingly, MSC treatment for four cycles, as a novel strategy, was provided to the patients. During the entire study period, only patient #2 experienced some adverse events including only one case of grade 1 diarrhea (<4 stools per day over baseline; mild increase in ostomy output compared to baseline output) and two cases of grade 3 high blood pressure (systolic blood pressure≧160 mmHg or diastolic blood pressure≧100 mmHg; medical intervention indicated; more than one drug or more intensive therapy than previously used indicated). In both patients, cytomegalovirus and BK virus PCR were observed with negative results during the study period. When one patient reported diarrhea, the results of infection-related studies were negative, and the serum tacrolimus trough level was 3.7 ng/mL. Except for those complications, there were no serious adverse events during the entire study period. On the other hand, renal function was stable through the final MSC infusion. Thereafter, it gradually deteriorated in each patient. Serum creatinine levels changed from 3.01 mg/dL to 7.81 mg/dL in patient #1 and from 2.87 mg/dL to 3.91 mg/dL in patient #2 (Figure 2). However, the patients did not experience graft failure, death, any infectious complications, or tumor development during the study period.

### 3.2. Changes in Subsets of CD4+ and CD8+ Cells in the PBMC Population

**Figure 3:**
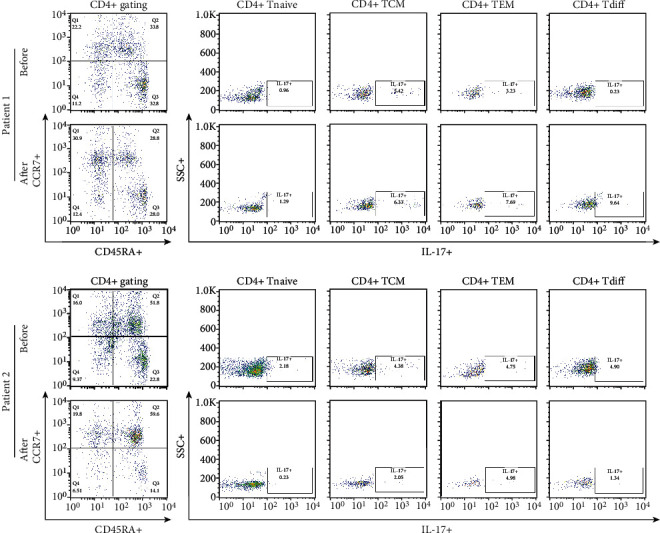
Comparison of CD4+ T cell subsets before and after MSC treatment. PBMCs were stained with anti-CD4 PE-Cy7-conjugated, anti-CD45RA FITC-conjugated, anti-CCR7 APC-conjugated, and anti-IL-17 PE-conjugated antibodies. CD4+ T cells were gated for further analysis. The proportions (%) of CD4+ T cells/lymphocytes, CD4+ Tnaive (CD45RA + CCR7+/CD4+ T cells), CD4+ TCM (CD45RA-CCR7+/CD4+ T cells), CD4+ TEM (CD45RA-CCR7-/CD4+ T cells), and CD4+ Tdiff (CD45RA + CCR7-/CD4+ T cells) in each patient were determined. After surface staining with anti-CD4 PE-Cy7-conjugated, anti-CD45RA ± FITC-conjugated, and anti-CCR7 APC-conjugated antibodies, analysis of IL-17 expression in CD4+ T cell subsets by intracellular flow cytometry was performed. The proportions (%) of IL-17+/CD4+ Tnaive (CD45RA + CCR7+/CD4+ T cells), IL-17+/CD4+ TCM (CD45RA-CCR7+/CD8+ T cells), IL-17+/CD4+ TEM (CD45RA-CCR7-/CD4+ T cells), and IL-17+/CD4+ Tdiff (CD45RA + CCR7-/CD4+ T cells) in each patient were determined. “Before” indicates the results at the start of MSC treatment, and “After” indicates the results at 3 months after the final MSC infusion. MSCs: mesenchymal stem cells; PBMCs: peripheral blood mononuclear cells.

**Figure 4:**
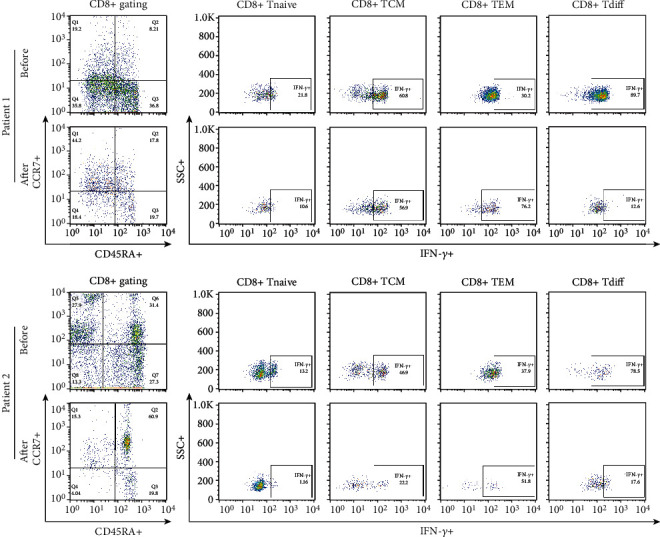
Comparison of CD8+ T cell subsets before and after MSC treatment. PBMCs were stained with anti-CD8 APC-conjugated, anti-CD45RA ± FITC-conjugated, anti-CCR7 APC-conjugated, and anti-IFN-*γ* antibodies. CD8+ T cells were gated for further analysis. The proportions (%) of CD8+ T cells/lymphocytes, CD8+ Tnaive (CD45RA + CCR7+/CD8+ T cells), CD8+ TCM (CD45RA-CCR7+/CD8+ T cells), CD8+ TEM (CD45RA-CCR7-/CD8+ T cells), and CD8+ Tdiff (CD45RA + CCR7-/CD8+ T cells) in each patient were determined. After surface staining with anti-CD8 APC-conjugated, anti-CD45RA ± FITC-conjugated, and anti-CCR7 APC-conjugated antibodies, analysis of IFN-*γ* expression in CD8+ T cell subsets by intracellular flow cytometry was performed. The proportions (%) of IFN-*γ*+/CD8+ Tnaive (CD45RA + CCR7+/CD8+ T cells), IFN-*γ*+/CD8+ TCM (CD45RA-CCR7+/CD8+ T cells), IFN-*γ*+/CD8+ TEM (CD45RA-CCR7-/CD8+ T cells), and IFN-*γ*+/CD8+ Tdiff (CD45RA + CCR7-/CD8+ T cells) in each patient were determined. “Before” indicates the results at the start of MSC treatment, and “After” indicates the results at 3 months after the final MSC infusion. MSCs: mesenchymal stem cells; PBMCs: peripheral blood mononuclear cells.

Total lymphocytes and the proportions of CD4+ cells and CD8+ cells among total lymphocytes did not differ after MSC treatment compared to the pretreatment period. In the analysis of the CD4+ T cell population and its subsets, the proportions of central memory, naïve, effector memory, and differentiated T cells among the total CD4+ T cell population determined by gating did not significantly differ between these periods (Figure 3). Additionally, in the analysis of the CD8+ T cell population and its subsets, the proportions of central memory, naïve, effector memory, and differentiated T cells among the total CD8+ T cell population determined by gating did not change (Figure 4).

### 3.3. Changes in Helper T Cells and Regulatory T Cells in the PBMC Population

**Figure 5:**
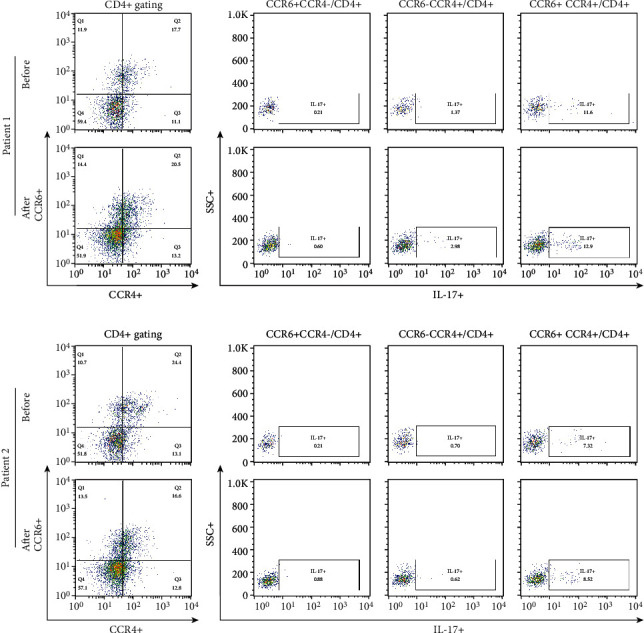
Comparison of CCR6 + CCR4-, CCR6-CCR4+, and CCR6 + CCR4+ subpopulations of CD4+ T lymphocytes before and after MSC treatment. PBMCs were stained with anti-CD4 PE-Cy7-conjugated, anti-CCR4 PE-conjugated, anti-CCR6 APC-conjugated, and anti-IL-17 FITC-conjugated antibodies. CD4+ T cells were gated for further analysis. The proportions (%) of CCR6-CCR4+/CD4+ T cells, CCR6 + CCR4-/CD4+ T cells, and CCR6 + CCR4+/CD4+ T cells in each patient were determined. After surface staining with anti-CD4, anti-CCR4, and anti-CCR6 mAbs, analysis of IL-17 expression in CD4+ T cell subsets by intracellular flow cytometry was performed. The proportion (%) of IL-17+/CCR6 + CCR4 + CD4+ T cells in each patient was determined. “Before” indicates the results at the start of MSC treatment, and “After” indicates the results at 3 months after the final MSC infusion. MSCs: mesenchymal stem cells; PBMCs: peripheral blood mononuclear cells.

**Figure 6:**
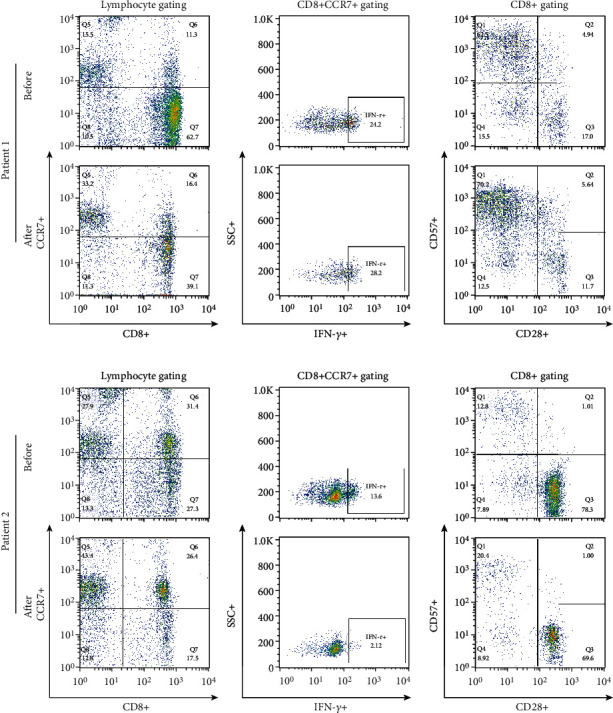
Comparison of CCR7 + CD8+ and effector T cells before and after MSC treatment. PBMCs were stained with anti-CD8 APC-conjugated, anti-CCR7 streptavidin Cy5.5-conjugated, anti-IFN-*γ* FITC-conjugated, anti-CD28 PE-conjugated, and anti-CD57 FITC-conjugated antibodies. The representative figure for the flow cytometric analysis of CCR7+ CD8+/lymphocytes and CD28-CD57+/CD8+ T cells is shown. “Before” indicates the results at the start of MSC treatment, and “After” indicates the results at 3 months after the final MSC infusion. MSCs: mesenchymal stem cells; PBMCs: peripheral blood mononuclear cells.

**Figure 7:**
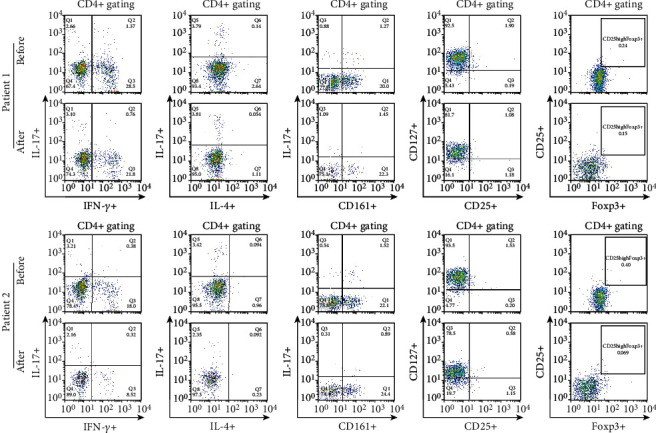
Comparison of Th1, Th2, Th17, and Treg subpopulations in the CD4+ T cell population before and after MSC treatment. PBMCs were stained with anti-CD4 PE-Cy7-conjugated, anti-CD25 APC-conjugated, anti-IFN-*γ* FITC-conjugated, anti-IL-17 PE-conjugated, anti-IL-4 APC-conjugated, anti-CD161 FITC-conjugated, and anti-Foxp3 FITC-conjugated antibodies. CD4+ T cells were gated for further analysis. The proportions (%) of IFN-*γ*+/CD4+ T cells, IL-17+/CD4+ T cells, IL-4+/CD4+ T cells, IL-17 + CD161+/CD4+ T cells, CD127lowCD25high/CD4+ T cells, and CD25 + FOXP3+/CD4+ T cells in each patient were determined. “Before” indicates the results at the start of MSC treatment, and “After” indicates the results at 3 months after the final MSC infusion. MSCs: mesenchymal stem cells; PBMCs: peripheral blood mononuclear cells.

The proportion of CCR6 + CCR4 + IL17+ cells among CD4+ T cells determined by gating was not different at 3 months after the last MSC treatment compared with the time before treatment (Figure 5). The percentages of IFN-*γ* + cells among CD8 + CCR7+ cells and CD28-CD57+ cells among CD8+ T cells did not change during the same period (Figure 6). The proportions of IL17+, IL-4+, and CD161+ cells among CD4+ T cells did not change between the two time points (Figure 7). The proportions of CD25highCD127low cells and CD25 + Foxp3+ cells among CD4+ T cells were not different at 3 months after the last MSC infusion compared to the pretreatment time point (Figure 7).

## 4. Discussion

Since CAMR is the most common cause of late allograft loss in KT, the establishment of a relevant therapeutic strategy for KTRs with CAMR is significant. Among the studies related to the application of MSCs in KT, reports of rejection treatment are very rare. In particular, only a few studies on CAMR can be found at ClinicalTrials.gov (NCT02563340 and NCT03585855), but patient outcomes remain unknown. Therefore, even if this study is a small pilot study, the results for MSC treatment of CAMR have some important implications. The purpose of this study was to confirm safety in CAMR patients receiving MSC treatment. The results clearly demonstrated that the patients did not experience serious adverse events except for mild diarrhea and blood pressure elevation. These findings are consistent with those of previous reports that MSCs exhibit favorable safety in KTRs [10–14]. Compared to previous studies which were conducted in patients early after transplantation, the most important aspect of this study was that multiple MSC treatments did not cause infectious complications or tumor development in patients with a large burden of immunosuppression created by long-term use of immunosuppressive agents.

In addition to safety, we evaluated the efficacy of MSC treatment. We expected improvement of graft function but graft function deteriorated during the 6 months after MSC treatment. Thus, one may argue that the clinical course of our cases after MSC treatment may be the natural course of CAMR, or MSC treatment may not be effective to control ongoing CAMR. The reason for unresponsiveness to MSC treatment is unclear but we speculate some possibilities. First, we included cases with advanced CAMR refractory to first-line therapy including rituximab. Second, there was no additional immunosuppression during MSC treatment. Third, the follow-up period was too short to evaluate the efficacy of MSC treatment. Hence, clinical trial of MSC treatment may be feasible in patients with early CAMR with superior renal function of our cases. In sequence, additional immunosuppression and long-term follow-up period may be needed to observe the efficacy of MSC treatment.

Finally, we evaluated the immunologic profile before and after MSC treatment. Since this study was conducted in patients exposed to rituximab, we identified changes in T cells to assess the immunologic effects of MSCs. It is well known that MSC administration concurrent with KT reduces the memory CD8+ T cell population at 12 months after KT [15] and the suppression of effector memory T cells may be the potential of CAMR treatment [16]. In addition, an increase in Treg cell numbers is an important factor to achieve immune tolerance, and a reduction in Treg cell numbers is closely associated with antibody-mediated rejection [17, 22]. Based on previous reports, we measured changes of subsets of CD4^+^ and CD8^+^ cells in the PBMCs before and after MSC treatment. We expected immunologic efficacy of MSCs on T cells but the results showed that memory T cells and Treg cells were not significantly different by MSC treatment (We could observe a slight decrease of IL-17+ cells and IFN-*γ* expression in memory T cells in patient #2). This finding suggests that MSC treatment may be ineffective in enhancing immunoregulatory function in patients with CAMR.

This study had some limitations. First, we did not compare changes in B cell-related markers owing to the effects of rituximab-based therapy before MSC infusion. Second, allograft biopsy after MSC treatment was not conducted. We considered repeated biopsy but did not include it because patients were reluctant to receive repeated biopsy with short-term interval.

## 5. Conclusions

There were no serious adverse events for six months after allogeneic MSC treatment in KTRs with CAMR refractory to rituximab and intravenous immunoglobulin. Further studies are needed to define the efficacy of MSC treatment in CAMR.

## Figures and Tables

**Table 1:** Features of the patients.

	Patient #1 (59/M)	Patient #2 (30/F)
KT age	44	24
Primary renal disease	HTN	MPGN
Comorbid disease	DM	HTN
Time between KT and MSC Tx	15 years 4 months	5 years 4 months
Height (cm)/weight (kg)/BMI (kg/m^2^) at time of MSC Tx	175.2/66.8/21.8	166.7/77.5/27.9
KDPI score	Unknown	-9
Donor age	23	31
Donor sex	Male	Male
Donor type	Deceased	Living
DSA	Unknown, but multiple strong DQs	Non-DSA with strong MFI in DQ
Previous AMR Tx	2 times	3 times
Time after last AMR Tx	4 months	2 months
Banff lesion scores for CAMR	G1 T0 I2 V0 AH3 PTC3 TI2 AAH3	G3 T1 I1 V0 AH0 PTC3 TI2 AAH0
	CG3 CT1 CI1 CV0 MM1 C4d3 i-IFTA1	CG3 CT1 CI1 CV0 MM1 C4d2 i-IFTA1

KT: kidney transplantation; HTN: hypertension; MPGN: membranoproliferative glomerulonephritis; DM: diabetes mellitus; MSC: mesenchymal stem cell; Tx: treatment; KDPI: kidney donor profile index; DSA: donor-specific antibody; AMR: antibody-mediated rejection; CAMR: chronic active antibody-mediated rejection; G: glomerulitis; T: tubulitis; I: interstitial inflammation; V: intimal arteritis; AH: arteriolar hyalinosis; PTC: peritubular capillaritis; TI: total inflammation; AAH: hyaline arteriolar thickening; CG: glomerular basement membrane double contours; CT: tubular atrophy; CI: interstitial fibrosis; CV: vascular fibrous intimal thickening; MM: mesangial matrix expansion; i-IFTA: inflammation in area of interstitial fibrosis and tubular atrophy.

## Data Availability

The data used to support the findings of this study are included in the article.

## Abbreviations

CAMR: Chronic active antibody-mediated rejection
KTRs: Kidney transplant recipients
MSCs: Mesenchymal stem cells
KT: Kidney transplantation
KFDA: Korean Food and Drug Administration
PBMCs: Peripheral blood mononuclear cells
Treg: Regulatory T cell.
